# Multilevel allometric modelling of maximum cardiac output, maximum arteriovenous oxygen difference, and peak oxygen uptake in 11–13-year-olds

**DOI:** 10.1007/s00421-020-04300-0

**Published:** 2020-01-10

**Authors:** Neil Armstrong, Jo Welsman

**Affiliations:** Children’s Health and Exercise Research Centre, University of Exeter, St Lukes Campus, Heavitree Road, Exeter, EX1 2LU UK

**Keywords:** Adolescents, Cardiorespiratory fitness, Cardiovascular variables, Children, Fat-free mass

## Abstract

**Purposes:**

To investigate longitudinally (1) the contribution of morphological covariates to explaining the development of maximum cardiac output (${\dot{\text{Q}}}$ max) and maximum arteriovenous oxygen difference (a-vO_2_ diff max), (2) sex differences in ${\dot{\text{Q}}}$ max and a-vO_2_ diff max once age, maturity status, and morphological covariates have been controlled for, and, (3) the contribution of concurrent changes in morphological and cardiovascular covariates to explaining the sex-specific development of peak oxygen uptake ($\dot{{V}}{\mathrm{O}}_{2}$).

**Methods:**

Fifty-one (32 boys) 11–13-year-olds had their peak $\dot{{V}}{\mathrm{O}}_{2}$, maximum heart rate (HR max), ${\dot{\text{Q}}}$ max, and a-vO_2_ diff max determined during treadmill running on three annual occasions. The data were analysed using multilevel allometric modelling.

**Results:**

There were no sex differences in HR max which was not significantly (*p* > 0.05) correlated with age, morphological variables, or peak $\dot{{V}}{\mathrm{O}}_{2}$. The best-fit models for ${\dot{\text{Q}}}$ max and a-vO_2_ diff max were with fat-free mass (FFM) as covariate with age, maturity status, and haemoglobin concentration not significant (*p* > 0.05). FFM was the dominant influence on the development of peak $\dot{{V}}{\mathrm{O}}_{2}$. With FFM controlled for, the introduction of either ${\dot{\text{Q}}}$ max or a-vO_2_ diff max to multilevel models of peak $\dot{{V}}{\mathrm{O}}_{2}$ resulted in significant (*p* < 0.05) additional contributions to explaining the sex difference.

**Conclusions:**

(1) With FFM controlled for, there were no sex differences in ${\dot{\text{Q}}}$ max or a-vO_2_ diff max, (2) FFM was the dominant influence on the development of peak $\dot{{V}}{\mathrm{O}}_{2}$, and (3) with FFM and either ${\dot{\text{Q}}}$ max or a-vO_2_ diff max controlled for, there remained an unresolved sex difference of ~ 4% in peak $\dot{{V}}{\mathrm{O}}_{2} .$

## Introduction

Peak oxygen uptake (${\dot{\text{V}}\text{O}}_{2}$), the highest ${\dot{\text{V}}\text{O}}_{2}$ elicited during an incremental exercise test to exhaustion, is internationally recognized as the ‘gold standard’ measure of youth cardiorespiratory fitness. Peak ${\dot{\text{V}}\text{O}}_{2}$ is probably the most researched physiological variable in paediatric exercise science (Falk et al. 2018), but the vast majority of investigations have been cross-sectional with data analyses clouded by inappropriate ratio scaling of peak ${\dot{\text{V}}\text{O}}_{2}$ with body mass (BM) and interpretation of youth peak ${\dot{\text{V}}\text{O}}_{2}$ in mL kg^−1^ min^−1^ (Welsman and Armstrong 2019). Even when interpreted appropriately, cross-sectional studies only provide a ‘snapshot’ of a continuous process and there are remarkably few rigorously analysed longitudinal studies of youth cardiorespiratory fitness. The influence of concurrent changes in morphological and physiological covariates on the development of peak ${\dot{\text{V}}\text{O}}_{2}$ during childhood and adolescence remains to be elucidated (Armstrong and McManus 2017).

Multilevel allometric modelling (Rasbash et al. 2018) allows the effects of age, maturity status, morphological covariates, and physiological covariates to be partitioned concurrently within an allometric framework to provide a sensitive interpretation of the development of peak $\dot{{V}}{\mathrm{O}}_{2}$. The application of multilevel allometric modelling has provided new insights into the role of morphological covariates in the development of cardiorespiratory fitness during childhood and adolescence (Armstrong and Welsman 2019a, b; Nevill et al. 1998). Recent studies have explored the influence of age- and maturity status-driven changes in BM and fat-free mass (FFM) on the development of peak ${\dot{\text{V}}\text{O}}_{2}$ from 10 to 18 years. Peak ${\dot{\text{V}}\text{O}}_{2}$ was demonstrated to be ergometer-specific but regardless of exercise modality (i.e., running or cycling) and in both sexes, with age and BM controlled for, maturity status made a positive contribution to explaining peak $\dot{{V}}{\mathrm{O}}_{2}$. However, when FFM was introduced to the multilevel allometric models in place of BM, the independent effects of maturity status were negated, and the models provided significantly (*p* < 0.05) better statistical fits to the data. However, even with FFM controlled for, an unexplained sex difference in peak ${\dot{\text{V}}\text{O}}_{2}$ remained with boys’ values significantly (*p* < 0.05) higher than those of girls (Armstrong and Welsman 2019a, b).

It is consistently reported that pulmonary ventilation does not limit the peak ${\dot{\text{V}}\text{O}}_{2}$ of healthy youth (Fawkner 2007; McManus and Armstrong 2017; Nixon 2018), so the unexplained sex difference in peak $\dot{{V}}{\mathrm{O}}_{2}$ is likely to be influenced by differences in cardiovascular variables at peak $\dot{{V}}{\mathrm{O}}_{2}$. Cardiovascular components of $\dot{{V}}{\mathrm{O}}_{2}$ are described by the Fick equation where $\dot{{V}}{\mathrm{O}}_{2}$ = cardiac output × arteriovenous oxygen difference and cardiac output is a function of heart rate and stroke volume. Both cross-sectional and longitudinal studies of heart rate at peak $\dot{{V}}{\mathrm{O}}_{2}$ (HR max) have reported that HR max is independent of age, maturity status, peak $\dot{{V}}{\mathrm{O}}_{2}$, and sex during childhood and early adolescence (Bailey et al. 1978; McNarry et al. 2014; Rowland et al. 1997b; Rutenfranz et al. 1981). In contrast, there are few cross-sectional studies of cardiac output at peak $\dot{{V}}{\mathrm{O}}_{2}$ (${\dot{\text{Q}}}$ max), stroke volume at peak $\dot{{V}}{\mathrm{O}}_{2}$ (SV max), or arteriovenous oxygen difference at peak $\dot{{V}}{\mathrm{O}}_{2}$ (a-vO_2_ diff max) (Rowland 2017; Turley 1997; Winsley 2007). Prior to the present study, no studies had investigated longitudinally the contribution of concurrent changes in morphological and cardiovascular variables to the development of peak $\dot{{V}}{\mathrm{O}}_{2}$.

Understanding the responses of cardiovascular variables to maximum exercise is limited by ethical and methodological challenges and no direct measurements of heathy children’s ${\dot{\text{Q}}}$ max or a-vO_2_ diff max have been reported. Several indirect methods of estimating ${\dot{\text{Q}}}$ during exercise have been developed and Doppler echocardiography, bioimpedance cardiography, and carbon dioxide (CO_2_) rebreathing have been demonstrated to be safe and reliable methods of estimating ${\dot{\text{Q}}}$ in paediatric exercise studies (Patterson et al. 1982; Warburton et al. 2008; Welsman et al. 2005). There are, however, no ‘gold standard’ reference values of ${\dot{\text{Q}}}$ max or a-vO_2_ diff max as all techniques suitable for use with healthy children are subject to varying degrees of error. Measures of cardiovascular variables should, therefore, only be compared within methodologies although trends are consistent across methodologies (Driscoll et al. 1989; Warburton and Breder 2018; Washington 1993).

Cross-sectional studies consistently show young adults to have higher absolute values of ${\dot{\text{Q}}}$ max (i.e., expressed in L·min^−1^) than children but when ${\dot{\text{Q}}}$ max is expressed in ratio with body surface area (BSA) (i.e. in L min^−1^ m^−2^) as the cardiac index values are similar (Nottin et al. 2002; Rowland et al. 1997a, 2000b). In childhood and early adolescence, cross-sectional studies suggest that boys’${\dot{\text{Q}}}$ max is typically larger than similar aged girls’${\dot{\text{Q}}}$ max and remains larger when expressed as the cardiac index (Rowland 2005; Vinet et al. 2003; Winsley 2007). However, ratio scaling ${\dot{\text{Q}}}$ max with BSA clouds understanding of its development.

Scaling ${\dot{\text{Q}}}$ max in ratio with BSA is convenient and traditional, but 70 years ago, Tanner (1949) unequivocally established that ratio scaling of cardiac data with BSA was fallacious. It has, subsequently, been demonstrated that the most appropriate method of scaling ${\dot{\text{Q}}}$ max is with a curvilinear allometric model (Batterham et al. 1997, 1999; Rowland 2017) and once it has been allometrically scaled with BSA neither age nor maturity status influence ${\dot{\text{Q}}}$ max (McNarry et al. 2014; Rowland 2017). ${\dot{\text{Q}}}$ max is, however, more closely matched to metabolic demand than body size and should be considered in relation to active muscle mass rather than BSA. Determining young people’s active muscle mass is experimentally challenging, and FFM has emerged as a pragmatic and appropriate morphological variable with which to scale ${\dot{\text{Q}}}$ max in developmental exercise physiology.

It has been compellingly argued that ${\dot{\text{Q}}}$ max is best expressed in relation to FFM raised to an empirically derived allometric scaling exponent calculated from the participants in a study (Batterham et al. 1997, 1999; Rowland 2017). In a well-designed cross-sectional study, Vinet et al. (2003) demonstrated that when ${\dot{\text{Q}}}$ max was expressed relative to FFM^0.76^, there were no longer ${\dot{\text{Q}}}$ max differences between 10 to 12-year-old boys and girls. However, appropriately modelled longitudinal studies are required to elucidate the development of ${\dot{\text{Q}}}$ max and its influence on the development of peak $\dot{{V}}{\mathrm{O}}_{2}$ during childhood and adolescence.

In a longitudinal investigation with the same participants as in the present study, we reported that with FFM controlled for, there was no significant (*p* > 0.05) sex difference in SV max. Moreover, with FFM controlled for, a ~ 5% sex difference in peak $\dot{{V}}{\mathrm{O}}_{2}$ was present and the introduction of SV max to the multilevel allometric model revealed that SV max made a significant (*p* < 0.05) additional contribution to explaining the development of peak $\dot{{V}}{\mathrm{O}}_{2}.$ However, a residual ~ 4% sex difference remained (Armstrong and Welsman 2019c).

There are few reports of a-vO_2_ diff max in children and early adolescents with data from girls particularly sparse. As a-vO_2_ diff max is normally calculated from ${\dot{\text{Q}}}$ max and peak $\dot{{V}}{\mathrm{O}}_{2}$, measures of a-vO_2_ diff max have similar limitations to those for ${\dot{\text{Q}}}$ max. Cross-study comparisons of data must, therefore, be made with caution. Within studies, a-vO_2_ diff max has consistently been reported to be higher in young adult men than in boys (Nottin et al. 2002; Rowland et al. 1997a), but data from studies focused on children and early adolescents indicate that a-vO_2_ diff max is not influenced by age (Gilliam et al. 1977; Rowland 2005; Yamaji and Miyashita 1977), sex (Obert et al. 2003; Rowland et al. 2000a; Vinet et al. 2003), or maturity status (McNarry et al. 2011, 2014; Rowland 2017). Significant associations between a-vO_2_ diff max and peak $\dot{{V}}{\mathrm{O}}_{2}$ have not been identified in children and adolescents (Rowland 2017; Rowland et al. 1999b).

In summary, changes in FFM have been demonstrated to be the dominant morphological influence on the development of peak $\dot{{V}}{\mathrm{O}}_{2}$ in both sexes (Armstrong and Welsman 2019a, b). SV max makes a small, additional contribution to FFM in explaining sex differences in the development of peak $\dot{{V}}{\mathrm{O}}_{2}$ (Armstrong and Welsman 2019c) and HR max appears to be independent of peak $\dot{{V}}{\mathrm{O}}_{2}$ and sex (Rowland 2017). The longitudinal development of ${\dot{\text{Q}}}$ max, and a-vO_2_ diff max and their influence on the development of peak $\dot{{V}}{\mathrm{O}}_{2}$ have not been rigorously explored using appropriate models in which covariates are partitioned concurrently within an allometric framework. The purposes of the present study are, therefore, to use multilevel allometric modelling to investigate in children and early adolescents: (1) the contribution of morphological covariates to explaining the development of ${\dot{\text{Q}}}$ max and a-vO_2_ diff max, (2) sex differences in ${\dot{\text{Q}}}$ max and a-vO_2_ diff max once age, maturity status, and morphological covariates have been controlled for, and (3) the contribution of concurrent changes in morphological and cardiovascular covariates to explaining the sex-specific development of peak $\dot{{V}}{\mathrm{O}}_{2}.$

## Methods

### Participants

Fifty-one (32 boys) 11–13-year-olds participating in a longitudinal study of cardiorespiratory fitness and short-term power output (Armstrong and Welsman 2019a, d) volunteered to have their maximum cardiovascular responses to exercise determined on three annual occasions. The SV max data have been published (Armstrong and Welsman 2019c), but complementary data on HR max, ${\dot{\text{Q}}}$ max, and a-vO_2_ diff max have not previously been analysed or reported.

### Experimental procedures

#### Determination of resting variables

Participants were well habituated to the laboratory environment, to the laboratory personnel, and to the experimental procedures. Age was computed from date of birth and date of test. Anthropometric measures were taken as described by the International Biological Programme (Weiner and Lourie 1981) and apparatus was calibrated according to the manufacturers’ instructions. BM was assessed using Avery balance scales (Avery, Birmingham, UK), stature was measured using a Holtain stadiometer (Holtain, Crmych, Dyfed, UK), and skinfold thicknesses over the triceps and subscapular regions were measured using Holtain skinfold callipers. Maturity status was visually assessed by the Research Centre nurse using the indices for pubic hair (PH) development described by Tanner (1962). FFM was estimated from skinfolds, BM, and maturity status using the youth-specific equations developed by Slaughter et al. (1988). Haemoglobin (Hb) concentration was determined as the mean value from duplicate fingertip blood samples which were immediately assayed using a Hemo Cue photometer (Clandon Scientific, Farnborough, UK).

#### Determination of exercise variables

Participants attended the Research Centre annually on two consecutive mornings to complete the required exercise protocols. Peak $\dot{{V}}{\mathrm{O}}_{2}$ and HR at peak $\dot{{V}}{\mathrm{O}}_{2}$ were determined on day one, and at a similar time, the following morning SV max was determined. All exercise tests were preceded by a standardized warm-up. Peak $\dot{{V}}{\mathrm{O}}_{2}$ was determined during a discontinuous, incremental exercise test to voluntary exhaustion on a motorized treadmill (Woodway, Cranlea Medical, Birmingham, UK). HR was monitored using an electrocardiograph (Rigel, Morden, UK) and expired respiratory gases were monitored continuously using an Oxycon Sigma on-line gas-analysis system (Cranlea Medical) which was calibrated prior to each test using gases of verified concentration and an appropriate range of flow rates using a Hans Rudolph calibration syringe (Cranlea Medical). The tests began at a treadmill belt speed of 1.94 m s^−1^ (7 km h^−1^) which was increased by 0.28 m s^−1^ (1 km h^−1^) every 3 min until a speed of 2.78 m s^−1^ (10 km h^−1^) was reached. Subsequently, belt speed was held constant and the gradient was incrementally increased by 2.5% every 3 min until voluntary exhaustion. A 1 min rest period separated the exercise stages. The highest 30 s $\dot{{V}}{\mathrm{O}}_{2}$ attained was accepted as a maximal index if clear signs of intense exertion (e.g., hyperpnea, facial flushing unsteady gait, and profuse sweating) were demonstrated and supported by a respiratory exchange ratio greater than 1.00 and an HR which was levelling-off over the final stages of the test at a value within 5% of the mean peak HR that we have previously reported for large groups of similar aged young people using the same test protocol (Armstrong et al. 1991). All participants reported in this study satisfied these criteria.

On the following morning, using the same apparatus including the ${\dot{\text{Q}}}$ determination facility of the Oxycon Sigma $\dot{{V}}{\mathrm{O}}_{2}$, HR, and ${\dot{\text{Q}}}$ were determined during the final minute of 3 min treadmill running at 2.50 m s^−1^ (9 km h^−1^). ${\dot{\text{Q}}}$ was determined using the CO_2_ rebreathing technique in accord with the methodology recommended by Jones (1997). The partial pressure of CO_2_ in arterial blood (PaCO_2_) was estimated from the end-tidal CO_2_. The partial pressure of mixed venous CO_2_ (PvCO_2_) was estimated from the rebreathing equilibrium. The downstream correction was applied to the partial pressure of the equilibration CO_2_ to adjust for alveolar to blood partial pressure differences. The CO_2_ content of venous and arterial blood was calculated from PvCO_2_ and PaCO_2_ using the McHardy curve adjusted for the effect of individual differences in Hb (McHardy et al. 1967). The volume of gas in the rebreathing bag was calculated to be 1.5 times the mean of three previous tidal breaths and the bag CO_2_ concentration, which varied from 9 to 13%, was calculated based on the $\dot{{V}}{\mathrm{O}}_{2}$ and the end-tidal partial pressure of CO_2_. The size of the rebreathing bag was selected individually to accommodate the gas volume but without being so large as to prevent appropriate CO_2_ equilibrium. Only tests which demonstrated a CO_2_ equilibrium were included in the data (Jones 1997).

### Data analysis

Data were stored and analysed using SPSS version 25 (IBM, SPSS statistics, Portsmouth, UK). In a preliminary study, we confirmed with these students that SV reaches its maximal value and plateaus during progressive treadmill running above moderate-exercise intensities. We reported no significant (*p* > 0.05) change (~ 2–3%) in SV running at 2.22 m s^−1^ (8 km h^−1^), 2.50 m s^−1^ (9 km h^−1^) and 2.78 m s^−1^ (10 km h^−1^) despite a significant (*p* < 0.05) ~ 16–17% increase in $\dot{{V}}{\mathrm{O}}_{2}$ (Armstrong and Welsman 2019c). SV as determined herein was, therefore, recorded as SV max. Individual values of ${\dot{\text{Q}}}$ max were determined by multiplying SV max by HR max and a-vO_2_ diff max was calculated by re-arranging the Fick equation (i.e., a-vO_2_ diff max = peak $\dot{{V}}{\mathrm{O}}_{2}$/${\dot{\text{Q}}}$ max). Longitudinal relationships between BM and FFM, respectively, and cardiovascular variables and between cardiovascular variables and peak $\dot{{V}}{\mathrm{O}}_{2}$ were graphed, and Pearson product-moment correlation coefficients computed with significance set at *p* < 0.05.

Longitudinal data were analysed using multilevel regression modelling (MLWin v3.02, Centre for Multilevel Modeling, University of Bristol, UK). In contrast to traditional methods that require a complete longitudinal data set, both the number of observations per individual and the temporal spacing of the observations can vary within a multilevel analysis. Longitudinal changes in ${\dot{\text{Q}}}$ max, a-vO_2_ diff max, and peak $\dot{{V}}{\mathrm{O}}_{2}$ were analysed using the multiplicative, allometric approach introduced to paediatric exercise physiology by Nevill et al. (1998) and implemented in subsequent longitudinal analyses of youth exercise performance (e.g., Armstrong and Welsman 2019a, b, c, d; Armstrong et al. 2019) as in Eqs. 1 and 2:

1 $$ y = {\text{ BM}}^{k} \cdot{\exp}\left( {a_{j} + b\cdot{\text{age}}} \right)\varepsilon_{ij} , $$

2 $$ y = {\text{ FFM}}^{k} \cdot{\exp}\left( {a_{j} + b\cdot{\text{age}}} \right)\varepsilon_{ij} , $$

where *y* = ${\dot{\text{Q}}}$ max, a-vO_2_ diff max, or peak $\dot{{V}}{\mathrm{O}}_{2}$.

Log transformation linearizes the model as in Eqs. 3 and 4 forming the starting point for analyses:

3 $$ {\log}_{{\text{e}}} y = k\cdot{\log}_{{\text{e}}} {\text{BM}} + a_{j} + b\cdot{\text{age}} + {\log}_{{\text{e}}} \left( {\varepsilon_{ij} } \right), $$

4 $$ {\log}_{{\text{e}}} y = k\cdot{\log}_{{\text{e}}} {\text{FFM}} + a_{j} + b\cdot{\text{age}} + {\log}_{{\text{e}}} \left( {\varepsilon_{ij} } \right), $$

where *y* = ${\dot{\text{Q}}}$ max, a-vO_2_ diff max, or peak $\dot{{V}}{\mathrm{O}}_{2}$.

All parameters were fixed with the exception of the constant (*a*) which was allowed to vary randomly at level 2 (between individuals) and the multiplicative error ratio (*ε*) which also varied randomly at level 1 (within individuals) as denoted by the subscripts *i* (level 1 variation) and *j* (level 2 variation). Age was centred on the group mean. Other factors associated with the dependent variable were explored either as additional covariates or through the calculation of dummy variables (e.g., setting the boys’ constant as baseline and investigating any departure from this for girls and setting PH stage 1 as the baseline from which effects of PH stages 2, 3, 4, and 5 could be explored) or interaction terms allowing for different relationships between covariates and sex to be examined.

Parameter estimates were considered significant (*p* < 0.05) where their value exceeded 2 × the standard error (SE). Where more than one model was investigated, a comparison of the goodness of fit of the different models was obtained from the change in the deviance statistic (− 2 × log-likelihood) with reference to the number of fitted parameters. The model with the smallest log-likelihood reflects that with the best fit for the same number of fitted parameters. Additional parameters contribute to improved fit from the change in the log-likelihood according to a chi-squared statistic for additional degrees of freedom added.

## Results

**Fig. 1:**
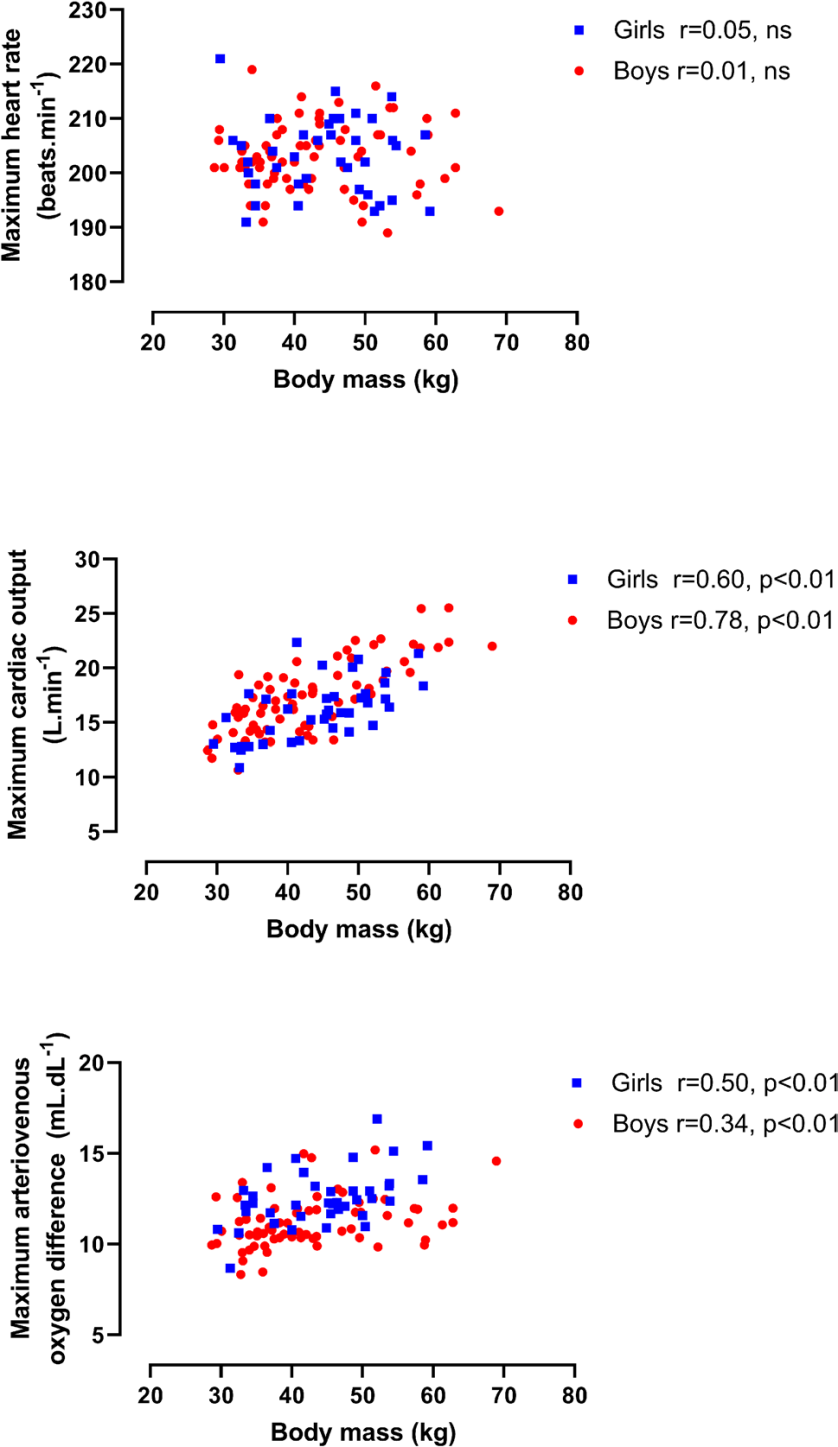
Maximum heart rate, maximum cardiac output, and maximum arteriovenous oxygen difference in relation to body mass

**Fig. 2:**
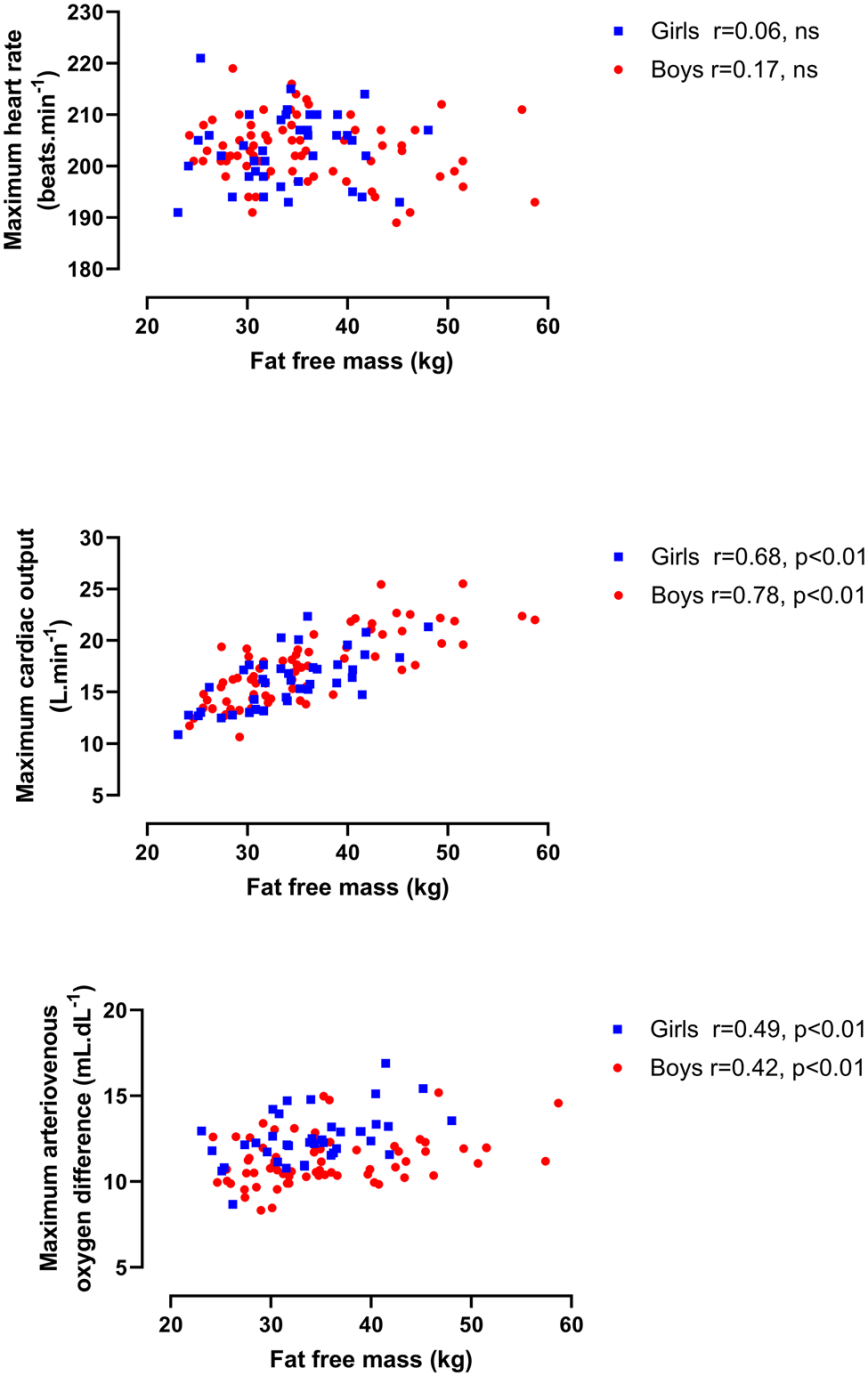
Maximum heart rate, maximum cardiac output, and maximum arteriovenous oxygen difference in relation to fat-free mass

**Fig. 3:**
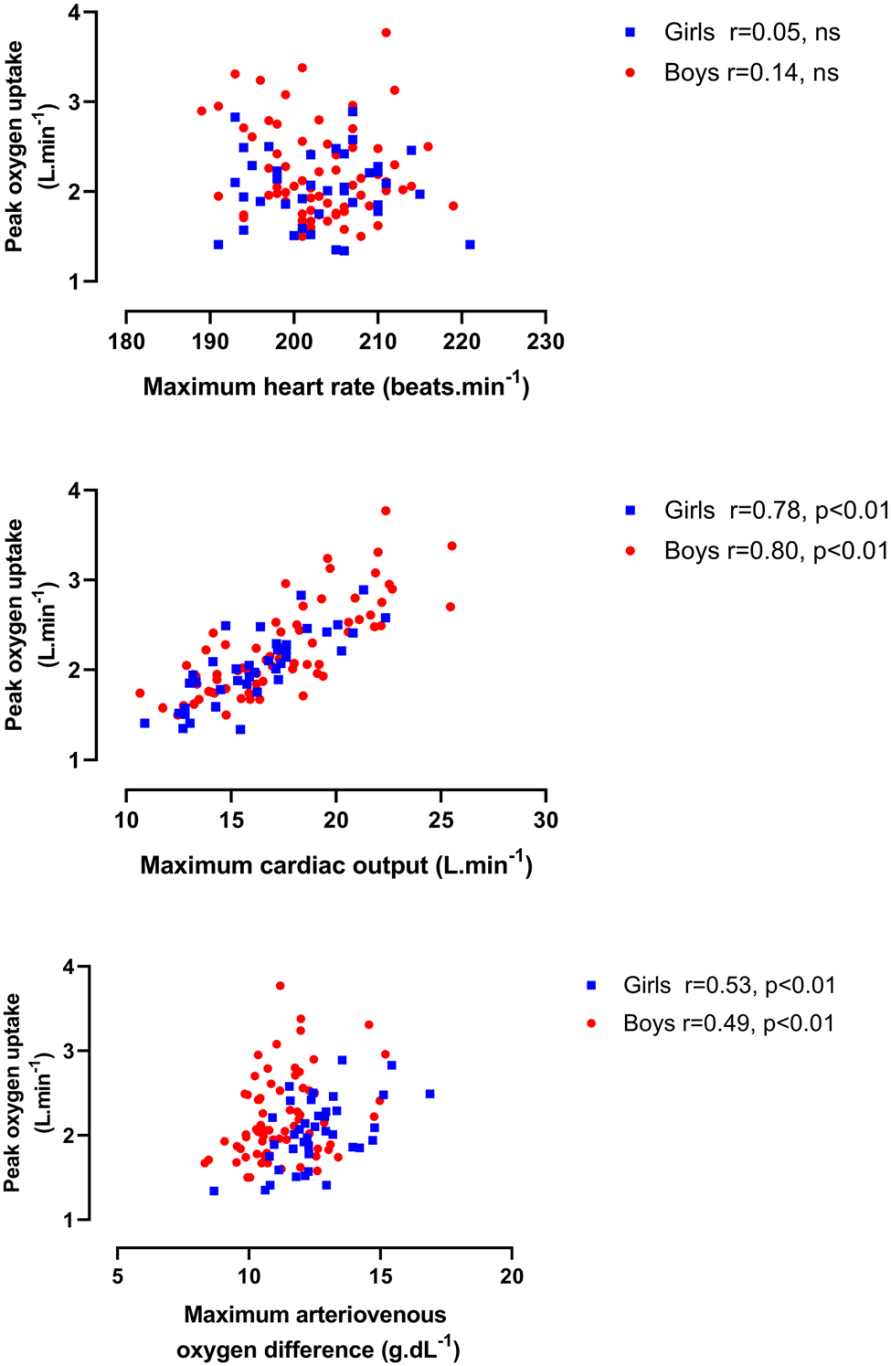
Peak oxygen uptake in relation to maximum heart rate, maximum cardiac output, and maximum arteriovenous oxygen difference

Longitudinal relationships by sex between HR max, ${\dot{\text{Q}}}$ max, and a-vO_2_ diff max, respectively, with BM and with FFM are illustrated in Figs. 1 and 2. ${\dot{\text{Q}}}$ max in boys was significantly (*p* < 0.01) and strongly correlated with both BM and FFM with moderate, significant (*p* < 0.01) correlations in girls. Significant (*p* < 0.01) but weak correlations between a-vO_2_ diff max and morphological variables were apparent in both sexes. HR max was not significantly (*p* > 0.05) correlated with BM or FFM, in either boys or girls. Figure 3 describes the relationships between HR max, ${\dot{\text{Q}}}$ max, and a-vO_2_ diff max, respectively, and peak $\dot{{V}}{\mathrm{O}}_{2}$, and shows no significant (*p* > 0.05) correlations between HR max and peak $\dot{{V}}{\mathrm{O}}_{2}$. ${\dot{\text{Q}}}$ max was significantly (*p* < 0.01) and strongly correlated with peak $\dot{{V}}{\mathrm{O}}_{2}$ in both sexes. a-vO_2_ diff max was also significantly (*p* < 0.01) correlated with peak $\dot{{V}}{\mathrm{O}}_{2}$ in both sexes, but with weak-to-moderate strength coefficients.

As HR max was not significantly (*p* > 0.05) related to BM, FFM, or peak $\dot{{V}}{\mathrm{O}}_{2,}$ the multilevel allometric analyses presented herein are focused on ${\dot{\text{Q}}}$ max and a-vO_2_ diff max. Table 1 presents multilevel allometric models for log_e_${\dot{\text{Q}}}$ max and log_e_ a-vO_2_ diff max with log_e_ BM or log_e_ FFM as covariates. In model 1.1, log_e_ BM was revealed as a significant (*p* < 0.05) covariate of log_e_${\dot{\text{Q}}}$ max with a significant (*p* < 0.05) negative sex term showing boys’ values to be higher than those of girls. In Model 1.2 with log_e_ FFM replacing log_e_ BM in the model, there was no significant (*p* > 0.05) sex difference in log_e_${\dot{\text{Q}}}$ max. Model 1.2 provided a significantly (*p* < 0.05) better statistical fit with log_e_${\dot{\text{Q}}}$ max increasing proportionally to FFM^0.69^. Model 1.3 shows log_e_ BM as a significant (*p* < 0.05) covariate of log_e_ a-vO_2_ diff max, but model 1.4 with log_e_ FFM replacing log_e_ BM provided a significantly (*p* < 0.05) better statistical fit. With either log_e_ BM or log_e_ FFM controlled for, there were no significant (*p* > 0.05) sex differences in log_e_ a-vO_2_ diff max. Once either log_e_ BM or log_e_ FFM had been controlled for, the introduction of age, maturity status, or Hb concentration into any model in Table 1 was not significant (*p* > 0.05).

**Table 1:** Multilevel allometric models for maximum cardiac output and maximum arteriovenous oxygen difference

Response	Model 1.1	SE	Model 1.2	SE	Model 1.3	SE	Model 1.4	SE
Fixed part	Log_e_ max cardiac output		Log_e_ max cardiac output		Log_e_ a-vO_2_ diff max**		Log_e_ a-vO_2_ diff max**	
Constant	0.460	0.228	0.363	0.212	1.574	0.222	1.517	0.210
Log_e_ body mass	0.634	0.061	–		0.258	0.059	–	
Log_e_ FFM*	–		0.691	0.060	–		0.289	0.059
Age	ns		ns		ns		ns	
Sex	− 0.084	0.032	ns		ns		ns	
Random part								
Level: id								
Var (cons)	0.008	0.002	0.006	0.002	0.004	0.002	0.004	0.002
Level: time								
Var (cons)	0.009	0.002	0.009	0.002	0.010	0.002	0.010	0.002
Units: id	51		51		51		51	
Units: time	110		110		110		110	
2*log-likelihood:	− 156.372		− 165.610		− 158.185		− 162.189	

Values are model estimates (standard error). With either body mass or fat-free mass controlled for, the introduction of age, maturity status, or haemoglobin concentration into any model in Table 1 was not significant (*p* > 0.05)

FFM* fat-free mass estimated from youth-specific equations (Slaughter et al. 1988), *a-vO*_*2*_ diff max** maximum arteriovenous oxygen difference; ns not significant (*p* > 0.05), *–* not entered

Table 2 describes multilevel allometric models for log_e_ peak $\dot{{V}}{\mathrm{O}}_{2}$. Model 2.1 shows, with log_e_ FFM controlled for, a significant (*p* < 0.05) sex difference of ~ 5% in log_e_ peak $\dot{{V}}{\mathrm{O}}_{2}$. Models 2.2 and 2.4 show with either log_e_${\dot{\text{Q}}}$ max or log_e_ a-vO_2_ diff max, respectively, controlled for, no significant (*p* > 0.05) sex differences in log_e_ peak $\dot{{V}}{\mathrm{O}}_{2}$. Model 2.1 is a significantly (*p* < 0.05) better statistical fit for log_e_ peak $\dot{{V}}{\mathrm{O}}_{2}$ than either Model 2.2 or 2.4. Model 2.3 shows that with log_e_ FFM controlled for, log_e_${\dot{\text{Q}}}$ max was an additional and significant (*p* < 0.05) covariate of log_e_ peak $\dot{{V}}{\mathrm{O}}_{2}$ with a significant (*p* < 0.05) sex difference in log_e_ peak $\dot{{V}}{\mathrm{O}}_{2}$ of ~ 4%. Model 2.3 provides a significantly (*p* < 0.05) better fit than both Model 2.1 and Model 2.2. Similarly, Model 2.5 shows that with log_e_ FFM controlled for, log_e_ a-vO_2_ diff max was an additional and significant (*p* < 0.05) covariate of log_e_ peak $\dot{{V}}{\mathrm{O}}_{2}$ with a significant (*p* < 0.05) sex difference in log_e_ peak $\dot{{V}}{\mathrm{O}}_{2}$ of ~ 4%. Model 2.5 provides a significantly (*p* < 0.05) better fit than both Model 2.1 and Model 2.4. The introduction of age, maturity status, or Hb concentration into any model in Table 2 was not significant (*p* > 0.05).

**Table 2:** Multilevel allometric models for peak oxygen uptake

	Model 2.1	SE	Model 2.2	SE	Model 2.3	SE	Model 2.4	SE	Model 2.5	SE
Response	Log_e_ peak oxygen uptake		Log_e_ peak oxygen uptake		Log_e_ peak oxygen uptake		Log_e_ peak oxygen uptake		Log_e_ peak oxygen uptake	
Fixed part										
cons	− 2.708	0.133	− 1.716	0.197	− 2.770	0.130	− 1.130	0.302	− 3.022	0.146
Log_e_ FFM*	0.980	0.037	–		0.869	0.053	–		0.916	0.038
Sex	− 0.046	0.019	ns		− 0.041	0.018	ns		− 0.044	0.019
Log_e_ max ${\dot{\text{Q}}}$**	–		0.876	0.070	0.161	0.057	–		–	
Log_e_ a-vO_2_ diff max***	–				–		0.738	0.119	0.212	0.052
Random part										
Level: id										
Var (cons)	0.003	0.001	0.006	0.002	0.002	0.001	0.020	0.005	0.003	0.001
Level: time										
Var (cons)	0.003	0.001	0.011	0.002	0.003	0.001	0.014	0.003	0.002	0.000
Units: id	51		51		51		51		51	
Units: time	110		110		110		110		110	
− 2*log-likelihood:	− 272.000		− 143.711		− 279.275		− 84.733		− 287.110	

Values are model estimates (standard error). With fat-free mass, ${\dot{\text{Q}}}$ max or a-vO_2_ diff max controlled for, the introduction of age, maturity status, or haemoglobin concentration into any model in Table 2 was not significant (*p* > 0.05). Model 2.1 is modelled from data on these participants reported and discussed in detail in Armstrong and Welsman (2019c)

FFM* fat-free mass estimated from youth-specific equations (Slaughter et al. 1988), $\dot{Q}$max** maximum cardiac output, *a-vO*_*2*_ diff max*** maximum arteriovenous oxygen difference, *–* not entered

## Discussion

Directly comparing values of ${\dot{\text{Q}}}$ max and a-vO_2_ diff max data across methodologies must be done with extreme caution (Warburton and Breder 2018), but the present data are in accord with values from cross-sectional studies which used CO_2_ rebreathing with similarly aged children and adolescents (Gilliam et al. 1977; Miyamura and Honda 1973: Yamaji and Miyashita 1977). Cross-sectional studies of similarly aged young people have reported HR max and a-vO_2_ diff max to be independent of sex but boys’ ${\dot{\text{Q}}}$ max to be higher than girls’ ${\dot{\text{Q}}}$ max (Armstrong et al. 1991; Miyamura and Honda 1973; Obert et al. 2003; Rowland et al. 2000a; Vinet et al. 2003; Winsley et al. 2009). However, growth and maturation are continuous processes driven by individual biological clocks and cross-sectional ‘snapshots’ of paediatric exercise data provide limited insights into developmental exercise physiology (Armstrong 2019; Baxter-Jones 2017; Malina et al. 2004). There are no longitudinal studies with which to compare the descriptive relationships described in Figs. 1, 2, and 3. The figures nicely describe the data, but as discussed earlier, provide few meaningful insights into the development of ${\dot{\text{Q}}}$ max, a-vO_2_ diff max, or peak $\dot{{V}}{\mathrm{O}}_{2}$. In contrast, multilevel allometric modelling describes the underlying mean response as individual growth trajectories are modelled.

Model 1.1 (Table 1) shows that ${\dot{\text{Q}}}$ max increased in proportion to BM^0.63^ with a significant (*p* < 0.05) sex difference of ~ 8%. However, ${\dot{\text{Q}}}$ max is dependent on metabolic demand and more closely related to the volume of metabolically active tissue than to total BM. Data indicate that cardiac dimensions are closely associated with FFM and suggestive of a potential relationship between skeletal and cardiac muscularity (Batterham et al. 1997, 1999; George et al. 1991). As quantifying actively contracting muscle mass during maximum exercise in childhood and adolescence is extremely challenging, FFM has become established as an appropriate surrogate marker of active muscle (Batterham et al. 1999; Rowland 2017; Vinet et al. 2003). Model 1.2 shows that ${\dot{\text{Q}}}$ max increases in proportion to FFM^0.69^ and provides a model with no significant (*p* > 0.05) sex differences and a significantly (*p* < 0.05) better fit than Model 1.1. With FFM controlled for, neither age nor maturity status were significant (*p* > 0.05) explanatory variables, but this may have been influenced by the limited age range and population of maturity stages in the present data. There are no comparative longitudinal data on cardiovascular variables, but it has been demonstrated that when FFM replaces BM in a multilevel modelling analysis of the development of peak $\dot{{V}}{\mathrm{O}}_{2}$ from 10 to 18 years, the effects of maturity status are negated in both boys and girls (Armstrong and Welsman 2019a).

In childhood and adolescence, HR max is independent of body size. ${\dot{\text{Q}}}$ max and SV max should, therefore, relate to morphological variables by similar exponents. This is the case with the present data with SV max increasing in relation to BM^0.64^ with a significant (*p* < 0.05) sex difference of ~ 9% and in relation to FFM^0.70^ with no significant (*p* > 0.05) sex difference. The FFM exponents of both ${\dot{\text{Q}}}$ max and SV max fall within the 95% confidence limits of previous cross-sectional studies (Rowland et al. 2000a; Vinet et al. 2003). (SV max models are not presented herein as multilevel allometric models of the development of the SV max of these participants have been analysed in Armstrong and Welsman 2019c).

Models 1.3 and 1.4 show that a-vO_2_ diff max increases in relation to BM^0.26^ and FFM^0.29^, but Model 1.4 with FFM as covariate presents a significantly (*p* < 0.05) better fit than Model 1.3. With either BM or FFM controlled for, age, maturity status, Hb concentration, and sex were not significant (*p* > 0.05). Comparative data on boys and girls are sparse, but there is no compelling evidence to support sex differences in a-vO_2_ diff max during childhood and early adolescence (Obert et al. 2003; Rowland 2005, 2017; Vinet et al. 2003). a-vO_2_ diff max represents the difference at peak $\dot{{V}}{\mathrm{O}}_{2}$ between the arterial oxygen content of blood approaching the muscles and the venous oxygen content as it leaves. Arterial oxygen content is primarily dependent on Hb concentration and, consistent with cross-sectional data on similar aged students from elsewhere (Armstrong et al. 1991; Obert et al. 2003; Vinet et al. 2003), Hb concentration was not a significant (*p* > 0.05) covariate in the present study. There is no persuasive empirical evidence to suggest sex differences in peripheral factors which are likely to influence venous oxygen content and, therefore, peak $\dot{{V}}{\mathrm{O}}_{2}$. Potential influences on muscle oxygen utilization include blood flow distribution, muscle capillarization, mitochondrial density, muscle fibre types, muscle activation, and muscle aerobic enzyme activity, but experimental exploration in developmental exercise physiology awaits the emergence and application of appropriate non-invasive technology (Armstrong et al. 2017; Dotan et al. 2012; Malina et al. 2004; Rowland 2005).

It has been demonstrated that age and maturity status-driven changes in FFM provide the most powerful morphological influence on peak $\dot{{V}}{\mathrm{O}}_{2}$ in 10–18 year-olds of both sexes and account for much of the increasing sex difference in the development of youth cardiorespiratory fitness (Armstrong and Welsman 2019a, b). Over the 10–18 years age range, FFM increases by ~ 105% and ~ 55% in boys and girls, respectively, and with BM controlled for, there is a ~ 16% sex difference in peak $\dot{{V}}{\mathrm{O}}_{2}$ which falls to ~ 10% with FFM controlled for, in place of BM (Armstrong 2019; Armstrong and Welsman 2019a). Increases in peak $\dot{{V}}{\mathrm{O}}_{2}$ during youth are driven by gains in either oxygen delivery to or oxygen utilization by the active muscles, or both. Growth and development of active muscle mass, reflected by FFM, not only enhance muscle oxygen utilization during exercise but, through the peripheral muscle pump, also augment venous return, boost ${\dot{\text{Q}}}$ max, and, therefore, increase oxygen delivery to the muscles (Armstrong and McManus 2017; Armstrong et al. 2017; Rowland 2017).

From 11 to 13 years, FFM rises on average, by ~ 28% and ~ 26% in boys and girls, respectively, but with wide individual variations as it is driven not only by chronological age but by the timing and tempo of biological maturation. FFM increases by ~ 60% and ~ 30% in boys and girls, respectively, from 1 year prior to 1 year post peak height velocity (Armstrong 2019; Baxter-Jones et al. 2003). In the present data set with children and early adolescents, there was a ~ 12% sex difference in peak $\dot{{V}}{\mathrm{O}}_{2}$ with BM controlled for and Model 2.1 (Table 2) illustrates that with sex-specific changes in FFM controlled for, there was a smaller but still significant (*p* < 0.05) ~ 5% sex difference in peak $\dot{{V}}{\mathrm{O}}_{2}$.

Model 2.2 shows that peak $\dot{{V}}{\mathrm{O}}_{2}$ increases in relation to ${\dot{\text{Q}}}$ max^0.88^ and Model 2.4 shows that peak $\dot{{V}}{\mathrm{O}}_{2}$ increases in relation to a-vO_2_ diff max^0.74^. In both cases, once either ${\dot{\text{Q}}}$ max or a-vO_2_ diff max were controlled for, there was no significant (*p* > 0.05) sex difference in peak $\dot{{V}}{\mathrm{O}}_{2}$. However, Model 2.1 with FFM controlled for shows a significant (*p* < 0.05) sex difference in the development of peak $\dot{{V}}{\mathrm{O}}_{2}$ and provides a significantly (*p* < 0.05) better statistical fit than either Model 2.2 with ${\dot{\text{Q}}}$ max controlled for, or Model 2.4 with a-vO_2_ diff max controlled for.

Models 2.3 and 2.5, respectively, show that with FFM controlled for, both ${\dot{\text{Q}}}$ max and a-vO_2_ diff max are additional, independent and significant (*p* > 0.05) covariates of peak $\dot{{V}}{\mathrm{O}}_{2}$ with, in each case, a significant (*p* < 0.05) ~ 4% sex difference in peak $\dot{{V}}{\mathrm{O}}_{2}.$ The models including FFM and either ${\dot{\text{Q}}}$ max (Model 2.3) or a-vO_2_ diff max (Model 2.5) provide significantly (*p* < 0.05) better statistical fits than Model 2.1. This finding agrees with our earlier publication in which with FFM controlled for, the introduction of SV max provided a significantly (*p* < 0.05) better fit model of peak $\dot{{V}}{\mathrm{O}}_{2}$ than FFM alone, with a similar ~ 4% sex difference (Armstrong and Welsman 2019c).

Collectively, the data show that age- and maturity status-driven changes in FFM are the dominant morphological influences on the development of 11–13-year-olds’ ${\dot{\text{Q}}}$ max and a-vO_2_ diff max, and that with FFM controlled for, there are no significant sex differences in either ${\dot{\text{Q}}}$ max or a-vO_2_ diff max. FFM is also the dominant influence on the development of peak $\dot{{V}}{\mathrm{O}}_{2}$ but, even with FFM controlled for, there remains a ~ 5% sex difference in peak $\dot{{V}}{\mathrm{O}}_{2}$. Concurrent changes in FFM and either ${\dot{\text{Q}}}$ max or a-vO_2_ diff max provide a better fit model of peak $\dot{{V}}{\mathrm{O}}_{2}$ than FFM alone, but a residual and unexplained ~ 4% sex difference in peak $\dot{{V}}{\mathrm{O}}_{2}$ remains.

## Strengths and limitations

A potential criticism of the present study is using CO_2_ rebreathing to monitor SV at ~ 83% (girls) and ~ 74% (boys) of peak $\dot{{V}}{\mathrm{O}}_{2},$ extrapolating it to represent SV max and using it with HR max to calculate ${\dot{\text{Q}}}$ max. However, this SV response is one of the most consistently observed responses in cardiac exercise physiology and has been reliably demonstrated in healthy, untrained children and adolescents using a range of methodologies including CO_2_ rebreathing (Rowland 2017; Warburton and Bredin 2018; Washington 1993). Values of exercise SV above ~ 50% of peak $\dot{{V}}{\mathrm{O}}_{2}$ have been reported to reflect SV max and be characteristic of individuals as well as group means (Rowland et al. 1999a, 2000c; Rowland 2005). A unique strength of the present study is that despite an increase in exercise intensity resulting in a rise in $\dot{{V}}{\mathrm{O}}_{2}$ of ~ 16–17%, a SV plateau within ~ 2–3% was demonstrated in all participants. Moreover, SV data were collected as close to peak $\dot{{V}}{\mathrm{O}}_{2}$ as reasonable for the young participants, many of whom found CO_2_ rebreathing unpleasant at high exercise intensities (Armstrong and Welsman 2019c).

The estimation of FFM from BM and skinfold thicknesses rather than its direct measurement using more sophisticated technology can be criticised, but this methodology is well established in paediatric exercise physiology (Slaughter et al. 1988) and ‘direct’ measures of the body fat of 12–14-year-olds have recently been shown to vary widely across laboratory techniques (Ferri-Morales et al. 2018). Regardless of assessment, FFM includes tissues not involved in exercise and ideally active muscle mass would be directly determined on each test occasion, but this is not currently feasible in paediatric exercise studies.

With three annual measurement occasions, the study only partially covers childhood and adolescence and potential effects of age and maturity status might have been limited by the small age range. The study does, however, provide for the first time longitudinal insights into the development of ${\dot{\text{Q}}}$ max and a-vO_2_ diff max and on their influence both individually and with concurrent changes in FFM, on the development of the peak $\dot{{V}}{\mathrm{O}}_{2}$ of healthy, untrained children, and early adolescents. Moreover, collectively, the present study and a parallel study on the same students (Armstrong and Welsman 2019c) provide insights into the development of all components of the Fick equation and their effect on the development of peak $\dot{{V}}{\mathrm{O}}_{2}$. A major strength of the study lies in using multiplicative allometric modelling analyses in which individual growth trajectories are modelled and morphological and cardiovascular covariates are partitioned concurrently within an allometric framework to provide sensitive interpretations of the development of, ${\dot{\text{Q}}}$ max, a-vO_2_ diff max, and peak $\dot{{V}}{\mathrm{O}}_{2}.$

## Conclusions

${\dot{\text{Q}}}$ max and a-vO_2_ diff max increase in proportion to FFM^0.69^ and FFM^0.29^, respectively. Once FFM has been controlled for, multilevel allometric models demonstrate that, in accord with SV max, there are no sex differences in either the ${\dot{\text{Q}}}$ max or a-vO_2_ diff max of children and early adolescents. Changes in age- and maturity status-driven FFM are the dominant influences on the development of peak $\dot{{V}}{\mathrm{O}}_{2}$, but with FFM controlled for, there remains a ~ 5% sex difference. With FFM controlled for, concurrent changes in either ${\dot{\text{Q}}}$ max or a-vO_2_ diff max make additional, independent and significant contributions to explaining the development of peak $\dot{{V}}{\mathrm{O}}_{2}$, but a residual ~ 4% sex difference remains unresolved in each case. The data clearly demonstrate that rigorous exploration of the development of cardiorespiratory fitness during growth and maturation requires longitudinal studies which include analyses of the influence of concurrent changes in both morphological and physiological covariates.

## Abbreviations

a-vO_2_ diff: Arteriovenous oxygen difference
BM: Body mass
BSA: Body surface area
CO_2_: Carbon dioxide
$${\dot{\text{Q}}}$$: Cardiac output
FFM: Fat-free mass
Hb: Haemoglobin
HR: Heart rate
max: Maximum
$$\dot{{V}}{\mathrm{O}}_{2}$$: Oxygen uptake
PaCO_2_: Partial pressure of CO_2_ in arterial blood
PvCO_2_: Partial pressure of mixed venous CO_2_
PH: Pubic hair
SV: Stroke volume
